# A comprehensive approach to stool donor screening for faecal microbiota transplantation in China

**DOI:** 10.1186/s12934-021-01705-0

**Published:** 2021-11-27

**Authors:** Jianquan He, Xingxiang He, Yonghui Ma, Luxi Yang, Haiming Fang, Shu Shang, Huping Xia, Guanghui Lian, Hailing Tang, Qizhi Wang, Junping Wang, Zhihui Lin, Jianbo Wen, Yuedong Liu, Chunbao Zhai, Wen Wang, Xueliang Jiang, Ji Xuan, Morong Liu, Shiyun Lu, Xuejun Li, Han Wang, Cong Ouyang, Man Cao, Aiqiang Lin, Bangzhou Zhang, Depei Wu, Ye Chen, Chuanxing Xiao

**Affiliations:** 1 School of Medicine, Xiamen University, Xiamen, China; 2 Department of Gastroenterology, The First Affiliated Hospital of Clinical Medicine of Guangdong Pharmaceutical University, Guangzhou, China; 3 Department of Gastroenterology and Hepatology, The Second Hospital of Anhui Medical Univerisity, Hefei, China; 4 Department of Gastroenterology, The Fifth People’s Hospital of Shenyang, Shenyang, China; 5 Anorectal Diagnosis and Treatment Center, The General Hospital of Xinjiang Military Region, Wulumuqi, China; 6 Department of Gastroenterology, Xiangya Hospital, Changsha, China; 7 Department of Gastroenterology, Xi’an Central Hospital, Xi’an, China; 8 Department of Gastroenterology, The First Affiliated Hospital of Bengbu Medical College, Bengbu, China; 9 Department of Gastroenterology, The Affiliated People’s Hospital of Shanxi Medical University, Taiyuan, China; 10 Department of Gastroenterology, Fujian Provincial Hospital, Fuzhou, China; 11 Department of Gastroenterology, Pingxiang People’s Hospital, Pingxiang, China; 12 Department of Gastroenterology, The Third Affiliated Hospital of Liaoning University of Traditional Chinese Medicine, Shenyang, China; 13 Department of Proctology, The Affiliated People’s Hospital of Shanxi Medical University, Taiyuan, China; 14 Department of Gastroenterology, 900th Hospital of PLA, Fuzhou, China; 15 Department of Gastroenterology, The Second Affiliated Hospital of Shandong University of Traditional Chinese Medicine, Jinan, China; 16 Department of Gastroenterology, Jinling Hospital, Nanjing, China; 17 Department of Gastroenterology, Affiliated Hospital of Zunyi Medical University, Zunyi, China; 18 Department of Gastroenterology, The Second Affiliated Hospital of Anhui University of Traditional Chinese Medicine, Hefei, China; 19 Xiamen Treatgut Biotechnology Co., Ltd., Xiamen, China; 20 National Clinical Research Center for Hematologic Diseases, The First Affiliated Hospital of Soochow University, Suzhou, China; 21 Department of Gastroenterology, Nanfang Hospital, Southern Medical University, Guangzhou, China

**Keywords:** Faecal microbiota transplantation, Microbiota evaluation, Donor selection, Metagenomics, Ethical issue

## Abstract

**Background:**

Faecal microbiota transplantation (FMT) is an effective therapy for recurrent *Clostridium difficile* infections and chronic gastrointestional infections. However, the risks of FMT and the selection process of suitable donors remain insufficiently characterized. The eligibility rate for screening, underlying microbial basis, and core ethical issues of stool donors for FMT are yet to be elucidated in China.

**Results:**

The potential stool donors were screened from December 2017 to December 2019 with the help of an online survey, clinical assessments, and stool and blood testing. Bioinformatics analyses were performed, and the composition and stability of gut microbiota in stool obtained from eligible donors were dynamically observed using metagenomics. Meanwhile, we build a donor microbial evaluation index (DoMEI) for stool donor screening. In the screening process, we also focused on ethical principles and requirements. Of the 2071 participants, 66 donors were selected via the screening process (3.19% success rate). Although there were significant differences in gut microbiota among donors, we found that the changes in the gut microbiota of the same donor were typically more stable than those between donors over time.

**Conclusions:**

DoMEI provides a potential reference index for regular stool donor re-evaluation. In this retrospective study, we summarised the donor recruitment and screening procedure ensuring the safety and tolerability for FMT in China. Based on the latest advances in this field, we carried out rigorous recommendation and method which can assist stool bank and clinicians to screen eligible stool donor for FMT.

**Supplementary Information:**

The online version contains supplementary material available at 10.1186/s12934-021-01705-0.

## Introduction

Faecal microbiota transplantation (FMT) is a highly effective therapy for recurrent *Clostridium difficile* infection (rCDI) [1]. Emerging evidence has consistently demonstrated that FMT can be used to treat other diseases such as inflammatory bowel disease (IBD), irritable bowel syndrome (IBS), slow transit constipation, hepatic encephalopathy, autism and metabolic syndrome [2]. Additionally, the clinical use of FMT in a variety of human diseases associated with perturbed gut microbiota has been increasing rapidly [3].

FMT is considered as an established form of therapy for rCDI that utilises the healthy donor stool. There is no doubt that the selection of reliable healthy donors is a critical success factor of FMT; however, recruiting and retaining stool donors is not easy [4]. Screening and determining the health status of a donor are generally accompanied by a series of assessments, including health questionnaires, clinical evaluation, stool testing, and blood testing [5]. The rigorous screening process and significant time commitment substantially decrease the qualification rate of donors. Additionally, the increasing clinical application and poorly regulated donor screening poses a risk of potentially transmitting infections. Due to invasive infections of antibiotic resistant *Escherichia coli* strain, one immunocompromised individual died after receiving investigational FMT [6]. In June 2019, the US Food and Drug Administration (FDA) issued a safety alert for the potential risk of serious adverse reactions or life-threatening infections with the use of FMT. Additionally, since December 2019, a newly identified coronavirus (severe acute respiratory syndrome coronavirus (SARS-CoV-2) has been rapidly spreading in China and more than 30 countries, which has immensely challenged FMT donor screenings [7]. Consequently, it is necessary to re-evaluate current donor screening practises and call for strict standards.

The stability of human gut community can be considered as a functional property, and can be defined by its ability to resist perturbation (resistance) and to return to an equilibrium state afterwards (resilience) [8]. Gut microbial health is also not a single static state but a dynamic equilibrium. Much of the gut microbiota diversity remains unexplained, although the environment, diet, host genetics, and early microbial exposure have been reported in some studies [9]. However, the high gut microbiota diversity is typically associated with temporal stability and health [10]. Although many studies focus on diseases which was accompanied with lack of gut microbiome diversity, a better understanding of the healthy microbiome of stool donors can help to thoroughly monitor the safety and efficacy of FMT.

Study has delineated the range of structural and functional configurations in healthy human microbiomes [11]. Even healthy individuals harbour an abundant and diverse microbial community and differ remarkably in the microbiota composition [12]. However, the variance in these gut communities over time within a stool donor remains poorly understood. Besides the microbiome-based donor selection, studies exploring the ethics of human microbiome research are also scarce, with “The Human Microbiome: Ethical, Legal and Social Concerns” being a notable exception. The 2017 thematic collection on ethical issues in FMT suggested that authorities must prioritize development of appropriate and effective regulation of FMT to safeguard patients and donors [13]. But, it did not discuss the ethical issues in depth about stool donor recruitment. In this study, we promoted a more rigorous screening and operation procedure and discussed three core ethical issues for donor screening to improve the safety and ethic of FMT.

## Results

### Stool donor screening results based on our recommendations

**Fig. 1:**
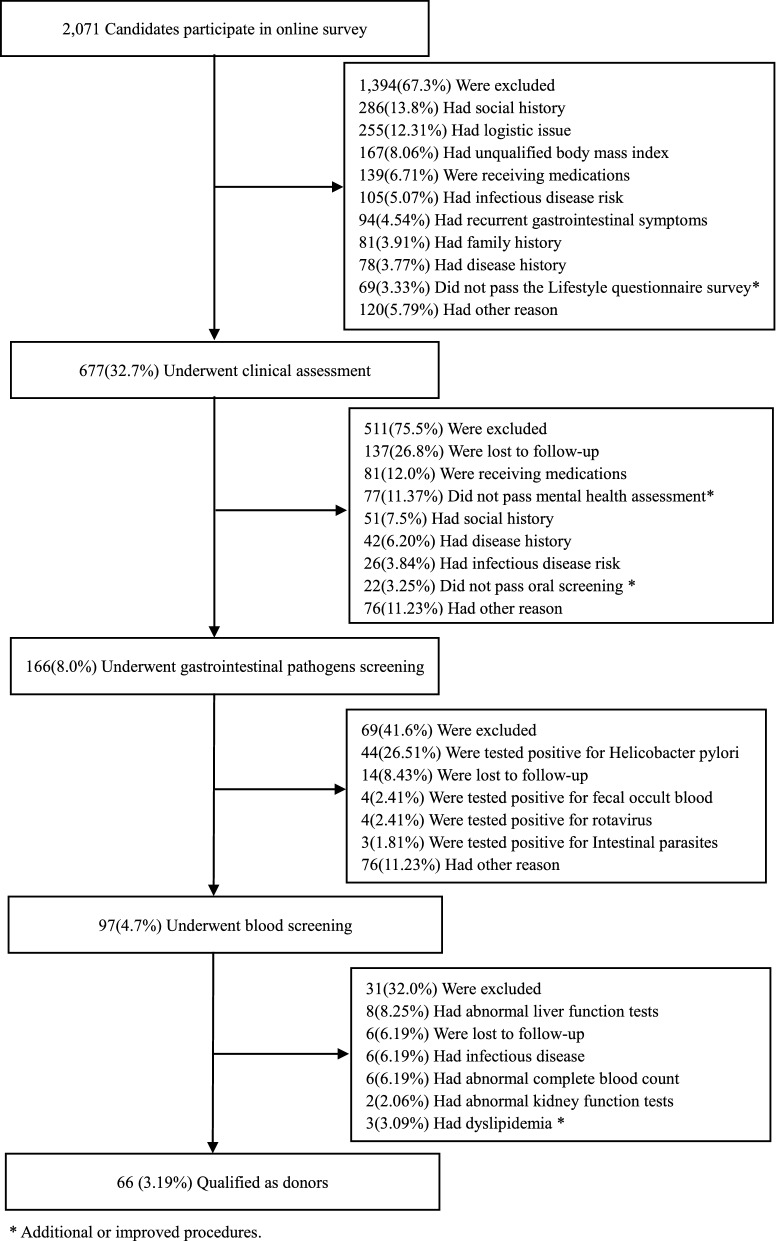
Flow program and outcomes of donor screening

From December 2017 to December 2019, our donor selection program evaluated a total of 2071 donor candidates, including 1403 from Xiamen and 668 from Guangzhou. First, during the pre-screening online survey stage, 1394 candidates (67.3%) were excluded mainly due to social history (286; 13.81%), logistic issues (255; 12.31%), and unqualified BMI (167; 8.06%). Meanwhile, 69 (3.33%) candidates did not pass the lifestyle questionnaire survey at this stage (Additional file 4: Table S1). Second, during the clinical assessment, an additional 511 (75.5%) were excluded, including 22 candidate donors for unqualified oral screening (each had caries, periodontal diseases, mucosal diseases, or oral cancer). A total of 98 candidates did not pass the mental health assessment (77; 11.37%) (Additional file 5: Table S2). Third, 69 (41.6%) of the remaining candidates were excluded during gastrointestinal pathogen screening, and the most prominent reason was positive testing for *H. pylori* (26.51%; Additional file 6: Table S3). A total of 31 participants (31.96%) were excluded after blood screening, including 14 who had abnormal hepatic serological indicators (eight with abnormal liver function tests and six with hepatitis B virus; Additional file 7: Table S4). In total, 66 participants qualified as stool donors, resulting in a 3.19% success rate (Fig. 1). As FDA has already reported the potential risk of transmission of SARS-CoV-2 via FMT, we added the testing of the stool donation or stool donor for SARS-CoV-2 virus in our new recommendations (Table 1).

**Table 1:** Summarized Donor Screening Recommendations

Initial Screening	Essential information and health questionnaire
Inclusion Criteria	^a^40 ≥ Age ≥ 18
	Body mass index (< 28 or > 18.5 kg/m^2^)
	Providing informed consent
	Keeping honesty and self-discipline
	Feeling well at the period of donation
	Children may donate with parental consent and child’s assent
Exclusion Criteria	High-risk behaviors
	Sexual practices associated with high risk of acquiring infectious diseases in last 12 months
	Known exposure with HIV, HAV, HBV, HCV infection in the last 12 months
	Intravenous drug use, incarceration, tattoo, piercing within previous 6 months
	Risk factors for variant Cruetzfeldt-Jakob disease
	Communicable disease
	History of HIV, HAV, HBV or HCV infection
	Any of the following in the previous 4 weeks: fever, vomiting, diarrhea or other symptoms of infection
	Any of the following in the previous 8 weeks: vaccinations, injections or contact with a recipient of the smallpox vaccine
	Any of the following in the previous 12 months: blood transfusion, accidental needle stick or blood exposure
	Travel within previous 6 months to areas of high risk of travelers’ diarrhea
	Close contacts with active gastrointestinal infection
	General medical illness or use of medication
	Social history (e.g., smoking, drinking, etc.)
	Receipt of antibiotics or PPI in the previous 3 months
	Using of medications (e.g., an experimental medicine, immunomodulatory therapy, chemotherapy, etc.)
	History of intrinsic gastrointestinal disease (e.g., inflammatory bowel disease, irritable bowel syndrome, chronic constipation, gastrointestinal malignancy, prior major gastrointestinal surgery, procedure, etc.)
	Strong family history of colorectal cancer
	Disease history (e.g., malignancy, malnutrition, chronic pain syndromes, neurologic or neurodevelopmental disorders, autoimmune or atopic illness, cardiovascular/metabolic disease, diabetes, hypertension, stroke, mental diseases, etc.)
	^a^Oral diseases (Caries, periodontal diseases, mucosal diseases or oral cancer)
	Others
	^a^Hamilton Anxiety Rating Scale (> 7 score), Hamilton Depression Rating Scale (> 7 score)
	^a^Lifestyle questionnaire survey (almost never exercise)
	Logistics issue (e.g., unable to donate regularly, distance to donor facility)
	Restrictive diet (e.g., gluten free diet)
	Abnormal vital sign (e.g., unexplained syncope)
Gastrointestinal pathogens testing	Routine tests
	Stool microscopy and culture, fecal occult blood test
	Intestinal parasites
	Fecal egg, cyst, microsporidia and parasites, Blastocystis hominis, Strongyloides stercoralis, Cyclospora, Isospora, Giardia, Cryptosporidium
	Intestinal pathogenic virus
	Rotavirus, Norovirus, Adenovirus
	Gastrointestinal pathogenic bacteria
	*Helicobacter pylori, C. difficile, Entamoeba, Shiga toxin with reflex to O157, Salmonella/Shigella, Vibrio cholera O1 and O139, Listeria monocytogenes, Escherichia coli O157 H7, Yersinia, Campylobacter*
	Intestinal drug-resistance bacteria
	Methicillin-Resistant *Staphylococcus aureus*, Vancomycin-Resistant Enterococci, Carbapenem-Resistant Enterobacteriaceae, Extended-Spectrum β-Lactamases bacteria
Blood testing	Routine tests
	Complete blood count, Liver function test, Renal function test, Blood glucose, Elevated C-Reactive Protein levels, Elevated dynamic ESR
	^a^Lipidemia test
	Infectious pathogen
	Treponema pallidum serology
	HTLV I/II, HAV, HBV, HCV, HEV, HIV
	^a^SARS-CoV-2 virus

^a^New recommendations

### Dynamic analysis of the microbial composition

#### Shotgun metagenomic evaluation

**Fig. 2:**
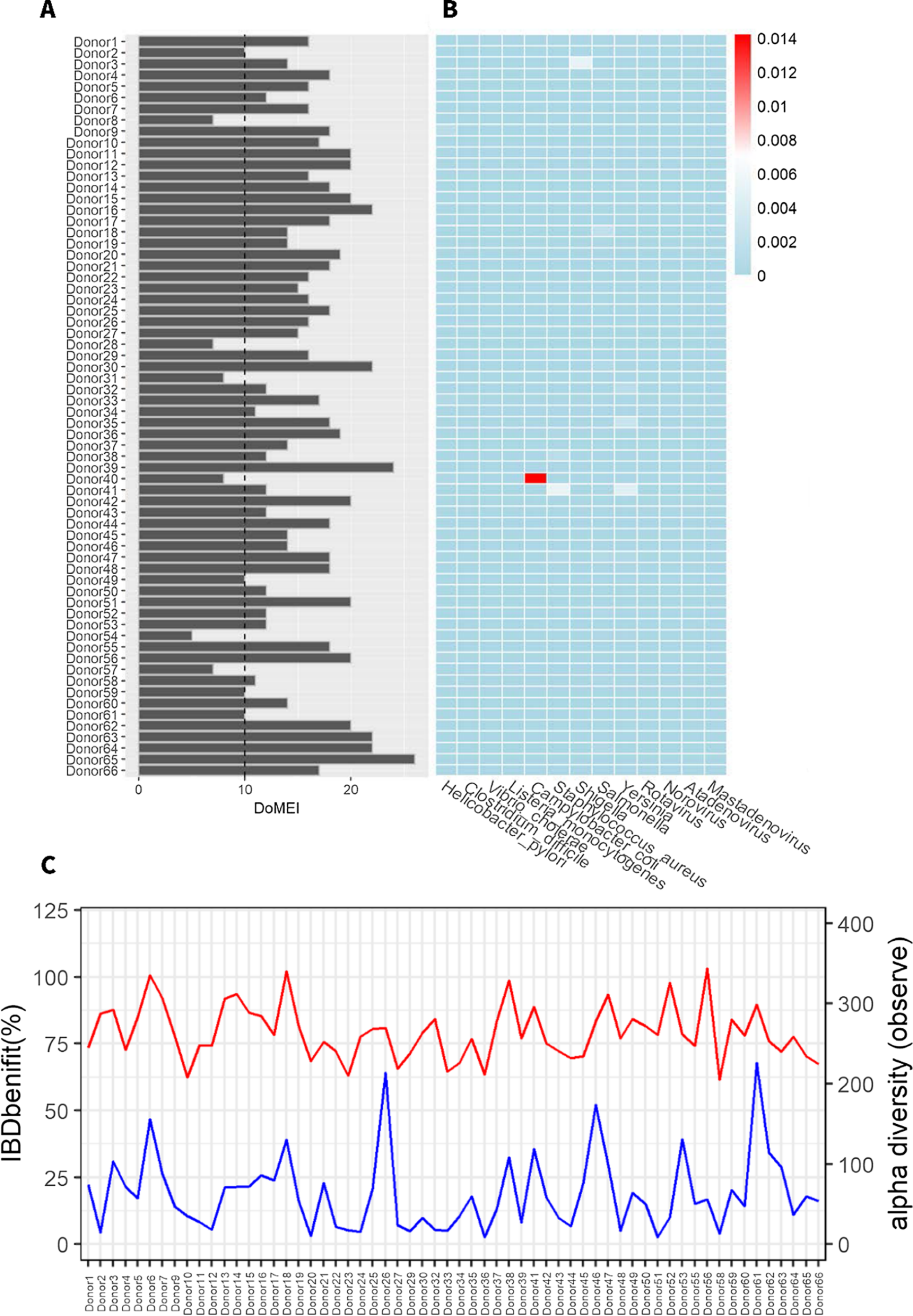
Shotgun metagenomics evaluation of donor microbiota. **A** DoMEI scores of candidate donors, based on 13 harmful bacteria, 16 beneficial bacteria, and microbial richness. DoMEI scores less than 10 is set as one of the signs of re-evaluated stool donors. **B** Relative abundances of pathogens of candidate donors. **C** Microbial richness (Chao 1, red) and relative abundances of beneficial taxa specific to IBD (blue) in qualified donors

A total of 427 Gb raw sequencing data (6.5 ± 0.9 Gb) were generated for the 66 potential donors. Due to the presence of gut harmful and beneficial microbial taxa, as well as microbial richness, low DoMEI scores (< 10 points) were observed in six donors (Donor8, Donor28, Donor31, Donor40, Donor54, and Donor57) (Fig. 2A), Furthermore, Donor40 was founded to carry high relative abundance of the clinical pathogen *Campylobacter coli* (0.014%) annotated by metagenomics (Fig. 2B). Due to the low DoMEI values, we re-evaluated online survey and clinical assessment of these six donors again. Donor8 and donor31 took traditional Chinese medicine before screening. Donor28 and donor57 had history of alcohol consumption. Donor54 took sleeping pills because of insomnia and irregular sleep patterns. Donor40 had recent history of diarrhea, but stool bacterium test was negative. The other 60 donors had high DoMEI scores (16.3 ± 3.6) and low relative abundances of pathogens (max = 0.005%). Finally, there were 60 donors who passed screening and were recruited [average age, 22.3 years (range, 18 to 30 years), 40% female, average BMI 22.58 kg/m^2^ (range, 20.20 to 25.81 kg/m^2^)]. Additionally, the qualified donors showed high gut microbial richness (264 ± 34, Fig. 2C) and various relative abundances of beneficial taxa for treating IBD, such as Ruminococcaceae and Lachnospiraceae.

### Stability and variation of donor gut microbial communities

**Fig. 3:**
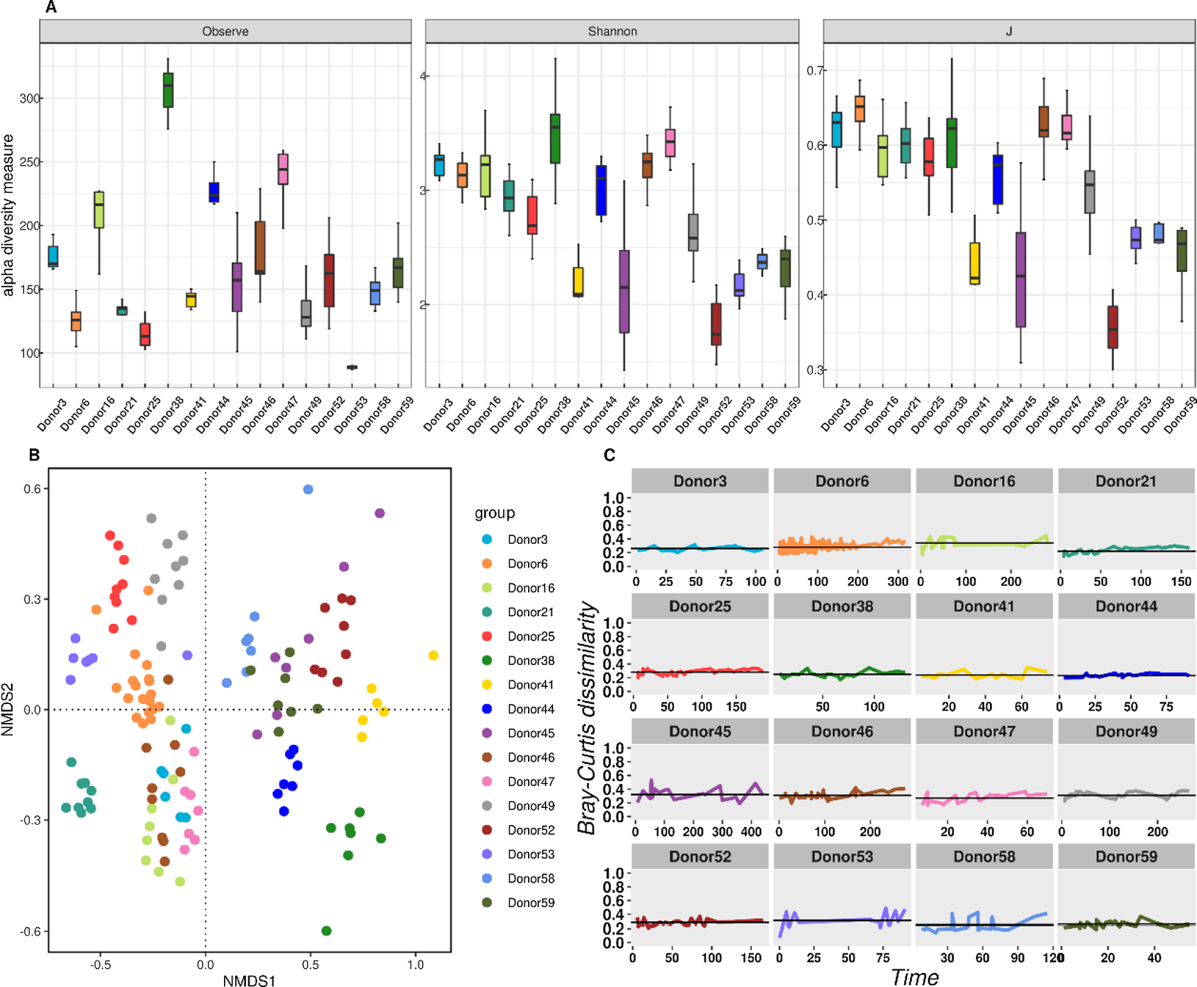
Donor gut bacterial variation and stability over time, based on 132 fecal samples consecutively collected from 16 frequent donors. **A** Differences in gut bacterial alpha-diversity indexes among donors: richness (*p* < 0.001), evenness (*p* < 0.001), Shannon (*p* < 0.001). **B** Non-metric multidimensional scaling (NMDS) ordinations of gut bacterial communities, based on Bray–Curtis dissimilarity (Stress = 0.17). **C** Changes in Bray–Curtis dissimilarity of gut microbiota within the same donor over time. Bray–Curtis dissimilarity between consecutive samples was plotted through time

A total of 132 samples from 16 frequent donors were sequenced to explore the stability of gut microbiota within the same and across donors (Additional file 8: Table S5). Significant differences in microbial diversity (Fig. 3A) and bacterial composition were observed among donors (Additional file 3: Fig. S1). For example, Donor 38 showed the highest richness and Shannon indices, Donor 52 showed the lowest richness, while Donor 53 showed the lowest Shannon and evenness. Additionally, *Bacteriodes* or *Prevotella* were dominantly present (Additional file 3: Fig. S1), revealing the donor enterotype characteristics. The overall communities were significantly different among donors (PERMANOVA, R^2^ = 0.7758, *p* = 0.001). However, it was apparent that samples collected from the same donor at different time points were generally close to each other in Non-metric multidimensional scaling (NMDS) ordination (Fig. 3B), indicating the stability of community structures.

To further quantify the community similarity over time, we calculated the Bray–Curtis dissimilarity from one sampling period to the next for each donor. The dissimilarity values within one donor ranged from 0.2 to 0.4, fluctuating around the mean value. In particular, some donors (Donor3, Donor25, and Donor44) remained quite stable with little variation over time (Fig. 3C), although Donor16 and Donor53 fluctuated in the early and late sampling periods. We further tested and found no significant differences in the dispersion of gut bacteria within each group (*p* = 0.543). These results indicate that the gut microbiota within each donor overtime was typically more stable than that between donors. Our findings also suggested that the faeces sampled and sequenced at present are likely to be similar to those taken after a few months.

## Discussion

In this study, we introduced a more stringent donor screening criterion for FMT in China, with 3.19% eligible donor rate, compared with the current 3–32% eligible donor rate across different stool banks with similar screening programs. For example, the largest stool bank (OpenBiome, Cambridge, MA) reported that only 3% of potential candidates successfully passed four necessary screening stages [4]. This is the first trial to track and compare the microbiota communities of stool donors. After sequencing and analysing bacterial community samples taken from 16 frequent donors at multiple time points by 16S rRNA gene sequencing, we observed the stability and variability of the bacterial communities across donors over the sampling period. Our results revealed a slight intra-donor variation in the gut microbiota. Indeed, gut microbiota has been reported to be stable in healthy adults in longitudinal cohort studies, with a subset of long-lived “resident” taxa that are rarely lost [14]. Therefore, due to the low qualification rate, recruiting stool donors was challenging in setting up a successful FMT program or stool bank.

Unlike blood donation, stool donation and FMT are not well known and accepted by the general public in China. The initial challenges for stool donor screening, including lack of knowledge of FMT and embarrassment of donation, are primary barriers to participating in the online pre-screening survey. Moreover, we observed that the largest proportion of donor candidates were excluded in the online survey (67.3% of all candidates), primarily due to social history (smoking and drinking), logistic issues, and unqualified BMI. Social history included smoking and drinking, which modify gut microbiota that provide a protective effect against gastrointestinal disorders or cancers [15].

Appropriate faecal donor age criteria remain inconsistent in other guidelines, studies, and stool banks. Generally, the recognised donor age ranged from 16 to 65 years [16], but we preferred to choose the age range from 18 to 40 years. On the one hand, the minimum donor age limit of 18 years was established as gastrointestinal microbial diversity plateaus [17] and individuals reach the legal age when they can provide valid informed consent in China. On the other hand, the referenced maximum donor age of 40 years was chosen as individuals above this age have gradually high prevalence rates of chronic diseases such as metabolic syndrome, hypertension, and diabetes in China [18]. It could possibly decrease productivity and increase the cost and schedule of stool donor screening. Obviously, long-term cost-effectiveness analyses of donors screening for FMT are necessarily needed in various age groups in the future. However, most of the other FMT donor selection program did not refuse 40 and over year-old perfectly healthy individual who has a proven health track record and meets the stool donor selection standard.

When this screening method was used, additional assessment scales were supplemented for the actual evaluation of lifestyle and mental health in the process of stool donor screening. The purpose of using the lifestyle questionnaire survey was to obtain information on health status, dietary habits, and physical activity. Diet plays a key role in shaping the gut microbiome, with several experiments demonstrating that dietary alterations can result in temporary and large microbial changes within 24 h [19]. Habitual diet, macronutrient composition, and dietary fibres have all been found to affect the human gastrointestinal microbiome [20]. Additionally, physical activity is associated with higher faecal bacterial alpha diversity and increased representation of some phyla (such as Firmicutes and Ruminococcaceae) and certain short-chain fatty acids in the faeces of healthy adults [21]. We excluded individuals that were overweight, indulged in unhealthy diets, and did not perform regular exercise from the online pre-screening survey. We recommend that it is necessary to focus on the diet structure and exercise activity of stool candidates. These habits, which are evaluated by various assessment scales, help to promote consistency and discipline in eligible donors.

Changes in gut microbiome composition have been reported in various mental diseases, including depression and anxiety [22], screening programs for mental disorders are indispensable in stool donor screening. In adolescents and young adults (aged between 15 and 39 years), mental health disorders such as anxiety disorders and depression are the major causes of disability [23]. The most common method of measurement is to employ rating scales, such as Hamilton Anxiety Rating Scale (HAMA) and Hamilton Depression Rating Scale (HAMD), in which the evaluation results have higher objectivity and reliability [24, 25]. In the clinical assessment stage of this study, both HAMA and HAMD excluded 77 (11.37%) participants who were undiagnosed with depression or anxiety through inquiry. These results suggest that HAMA and HAMD are recommended for the evaluation of mental disease for future clinical screening of stool donors.

We excluded FMT donors that might have carried any gastrointestinal pathogens. The rate of *H. pylori* infections in China are relatively high (40–60%), especially in young people, compared with other developed countries. *H. pylori* infections are closely related to the occurrence of gastric cancer; however, most infected people do not experience symptoms or complications. With this high incidence in China, *H. pylori* may be the preferred stool donor screening test for gastrointestinal pathogens. In our online pre-screening survey, we found that 44 of 166 participants tested positive for *H. pylori*.

Substantial evidence has verified that SARS-CoV-2 can be found in faeces by viral detection in biopsy specimens and stool [26]. The isolation of infectious SARS-CoV-2 viruses from stool samples of COVID-19 patients has directly proven the high possibility of the faecal-oral route of transmission [27]. To avoid the risk of faecal transmission, an international expert panel has recommended some temporary precautions, such as checking the donor’s history of travel to regions of outbreak, screening close contacts who were or are suspected of being infected, and quarantining donor stools for 30 days before use [7]. Another question for consideration is a group of asymptomatically infected individuals, which may propagate the virus and impede stool donor screening. Hence, we recommend tests for throat swab nucleic acid of COVID-19 before all the stool donations. Although such measures increase the cost and decrease productivity, they are worth conducting to ensure safe practices.

Beyond the clinical examination and faecal pathogen cultivation, we further extended the assessment of donors to include wider faecal microbiota and microbial richness using shotgun metagenomics for each candidate donor. Understanding and extending the assessment of donor community composition for FMT has been appealed recently. In practice, we built and applied the scoring DoMEI and pathogen assessments for all candidate donors since 2017, taking into consideration the published metagenomic data of donors and healthy subjects. It is necessary to accept re-evaluation when candidate donors who had low DoMEI scores (<10 points) or a high abundance of pathogens (>0.01%). Thanks to DoMEI scores, we found six candidates had different influencing factors on faecal microbiota and microbial richness. For example, the Donor 40 had a quite low DoMEI score (8 points) and also carried a relatively high abundance of pathogen *Campylobacter coli* (0.014%). *Campylobacter* has been the most commonly reported major human bacterial enteropathogen since 2005 in the European Union. When transmitted to humans, *Campylobacter coli* may particular cause gastroenteritis [28]. This indicates that DoMEI could be used as a reference index for stool donor re-evaluation, although it cannot be used as an exclusion criteria right now. Additionally, there are characteristic donor stool bacterial community associated with improved FMT efficacy, such as balanced constitution of Bacteroidetes versus Firmicutes [29], low level of *Fusobacterium* and *Ruminococcus gnavus* [30], high relative abundance of *Akkermansia muciniphila*, *unclassified Ruminococcaceae*, *Ruminococcus spp* and *Bifidobacterium* [31, 32]. In this study, all qualified donors showed high gut microbial richness, an important sign associated with better outcomes [1, 33]. A subset of the qualified donors displayed high relative abundances of Ruminococcaceae and Lachnospiraceae that can produce butyrate and are beneficial for FMT to treat IBD [33].

Our results showed little intra-donor variation of gut microbiota, and supported the view that a single assessment of faecal microbiota of donors is likely sufficient to represent the microbial compositions in subsequent months [1]. Alternatively, the differences among donors were much greater than the variations within the same donor, similar to observations in healthy subjects [34]. This is because there are distinct characteristics among donors, and we will be able to select an appropriate donor by developing algorithms to match recipients and donors based on their gut microbiota as well as factors such as diseases, gender, age, region, diets, and ethnicities [35]. With the donor-recipient matching algorithms, it is feasible to create a donor list for a specific recipient before FMT to achieve better efficacy and precision FMT. In spite of the clinically safety and effectiveness of FMT, researchers and clinician could not ignore the limitation of FMT owing to the risk of undesirable outcomes, disease transmission and uncertain effects of immune system on the recipients. Therefore, future studies to evaluate FMT to reduce risk of these conditions should clearly describe the donor recruitment and screening strategies. Aside from standardized and rigorous donor screening, it is essential to set up the FMT registry, adverse events monitoring, follow-up complications and clinical outcomes.

The accompanying ethical, social, and regulatory challenges must be addressed as part of an appropriate regulatory policy response to FMT to foster its safe and effective implementation. Concerns have been raised that whether a donor’s religious background, particularly where this entails special dietary requirements, should be considered when selecting and screening donors and allocating faecal microbiota to recipients [36]. For example, Muslim patients might have a strong objection against FMT from non-Muslim donors. Alternatively, vegan parents might oppose FMT for their child from non-vegan donors because they want to raise their child to comply with the same lifestyle [37]. Additionally, most donor screening guidelines require personal and private information on the donors’ health status, BMI, sexual activity, travel activity, family history, and transmissible diseases. The breaches of personal privacy could have detrimental implications for employment, education, and medical insurance. Identifying the donor details with in-house number could be a valuable method to solve confidentiality issue in FMT. Physicians and scientists need to be cautious when handling this information to avoid labelling some people as “risky” to some disease, especially in societies where social stigma is associated with mental illness and neurological disorders. It is of particular importance to examine existing regulations and protection to protect people from discrimination based on their microbiome, and then adjust or develop microbiome-specific regulations.

Increasing studies in human microbiome research have demonstrated that personal microbiomes contain enough distinguishing features to identify an individual over time based on their past travel experiences and sexual habits [38]. A notable concern is the association between the human microbiome and disease susceptibility, such as IBD, multiple sclerosis, and colon cancer. It is important to note that there is still a lack of scientific consensus on the validity of any conclusive results regarding undisclosed identification of individuals or links to disorders [39]. When human microbiome data recovered from stool donation is combined with human DNA data and other types of information, this could even generate “unprecedented personal-revealing information of a new magnitude” [40] far more than any other organ donation could achieve. An appropriate financial incentive for a stool donor is widely accepted in some stool banks. For example, OpenBiome in US offers up to $40 per stool donation, while some medical research centres provide $46 for each donation. Lastly, the level of compensation needs to strike a balance between three factors: incentive to recruit and retain donors, compensation for donor’s effort, and avoiding undue inducement of people from low socio-economic backgrounds [41]. The amount of compensation should also take the legal framework and social context in specific societies into consideration. To the best of our knowledge, we are the first to attempt incorporating microbial metagenomic assessment into donor evaluation for FMT studies, despite no clear agreement on selecting donors based on microbial parameters [42]. As increasing findings and sequencing data have been publicly available over time, the DoMEI scoring scheme should be further updated. In particular, we are planning to include wider microbial taxa of bacteria, fungi, and viruses (bacteriophage) at finer taxonomy levels and more diversity metrics, to consider functional features (SCFA production, bile salt metabolism, antibiotics-resistance) [33], and to optimise the scoring weight. Furthermore, other aspects, including faecal metabolites (SCFA), should also be considered to improve donor selection [43].

## Materials and methods

### Stool donor screening for FMT

#### Online pre-screening survey

Interested donors were recruited via local and internet media (public web platform, paper, local news) in Xiamen and Guangzhou, China. Donor candidates filled out an online pre-screening form to document the general health information, such as health status, occupation, disease history, medication history, family history, and risk of infectious disease. Empirically, it is suggested that potential FMT donors should be ≥ 18 and ≤ 40 years old and have a body mass index (BMI) between > 18.5 and < 28 kg/m^2^. Individuals who were active smokers and drinkers were excluded because these factors perturb the gut microbiome [44]. As it is important to limit the time of collection and preparation of faeces to preserve as many anaerobes as possible, donors were requested to live in the local region and defaecate in the appointed clean rooms. We included an additional lifestyle questionnaire survey, which has not been used in other reported methods. Using the data from this survey (Additional file 1: Appendix S1), we evaluated the temperament, physical activity, and dietary habits of donor candidates, preferring those candidates with the best health status and an enthusiastic and self-disciplined personality.

### In-person evaluation and clinical assessment

This evaluation was performed by a trained physician and nurse and supervised by a senior internal medicine specialist (Additional file 2: Appendix S2). The exclusion criteria in this assessment included atopic, allergic, gastrointestinal, autoimmune, metabolic, neurologic, and psychiatric conditions, as these are known to be associated with an abnormal intestinal microbiome profile. A positive response to a history of chronic pain syndromes, malignancy, receiving growth hormone or receiving an experimental medicine resulted in exclusion from further consideration as a donor. Our screening was inclusive of oral screening as an increasing number of diseases are associated with oral microbiomes [45]. Caries, periodontal diseases, mucosal diseases, and oral cancer were rejected. These assessments were conducted to exclude risk factors for potential microbiome-perturbated conditions and transmissible diseases. Additionally, all donor candidates were also assessed based on the Hamilton Anxiety Rating Scale (HAMA) and Hamilton Depression Rating Scale (HAMD). The inclusion and exclusion criteria are listed in Table 1. With the help of these tests, we sought to improve our screening program to incorporate subjective and scaled assessments consistently.

### Laboratory screening

Mandatory stool and blood screening of suitable donors was performed four weeks before donation. To ensure the suitability for inclusion as a donor during the test, laboratory screening was repeated regularly. Periodic testing typically required the donor stool and blood to be tested for all potential pathogens of concern at least every eight weeks (Table 1). A new donor sample of faeces was frozen and quarantined until all tests were confirmed to be negative. HIV testing was performed two weeks after the last donation was received. Stool samples of donors were only cleared for use if the test was negative.

### Ethical principles and requirements in donor selection and screening

We documented all the necessary health records including general information, online survey, clinical evaluation report, stool and blood test reports, and informed consent and financial incentives records, which conformed to the ethical principles. The issue of informed consent for FMT has become particularly prominent because there is limited information on the potential side effects of FMT, especially related to mental health-associated microbiota, including depression, anxiety, and mood. Studies on the informed consent of patients who accept FMT treatment or subjects participating in human microbiome research highlight the difficulties in identifying and explaining the potential risks and benefits of FMT [46], as well as the vulnerability of recipients [13]. We offered $31 to stool donors for each donation.

### Collection and preparation of faeces

On the day of donation, donors filled out a questionnaire that assessed their general health, new gastrointestinal symptoms, stool pattern/frequency, use of antibiotics, travel history, and sexual behaviour. We recommend that a health-related questionnaire be provided at the time of each stool donation. After passing the questionnaire, we gave donors a sterile plastic box that could be opened over a toilet to collect the stool. Cooler boxes and bags could be used so that the samples could be delivered to the specified site within 1 h after defecation. Stool samples were stored for up to 4 h at 4 °C in an air-tight box.

A minimum amount of 100 g of fresh stool sample was required for each donation. Fresh stool (25%) should be blended with normal saline (60%) and pharmaceutical grade glycerol (15%), and placed in an automatic stirring and separation machine (Treatgut Biotechnology Co., Ltd.). This machine is performed in 4 °C in order to preserve the microbial samples while processing the faeces. The process of blending was performed in an anaerobic chamber to prevent obligate anaerobic bacteria from exposure to oxygen. After blending, the stool mixture was aliquoted into individual sterile cryotolerant pots in a biological safety cabinet. The specifications, appearance, quantity, and weights of all the products were checked thoroughly. All samples were immediately frozen at − 80 °C. We carefully labelled each sample with the information and the date. The label is identified by a special number which only accessible by the authority and can be traced in the event of potential illness developing upon received by the recipients.

### Faecal DNA extraction, library generation, and sequencing

Faecal samples from each donor were collected to obtain the gut microbiota information. Total DNA was extracted from the samples weighing 0.25 g, using the QIAamp Fast DNA Stool Mini Kit (Qiagen, CA, USA). The resulting DNA yield and quality were checked with Qubit^®^ dsDNA HS Assay kit (Thermo Fisher Scientific, MA, USA). For shotgun metagenomics, DNA was fragmented to an average insert size of 350 bp using a Bioruptor NGS sonicator (Diagenode, BE) and further selected using VAHTS™ DNA Clean Beads (Vazyme, NJ, China). Metagenomic libraries were generated using the NEBNext^®^ Ultra™ II DNA Library Prep Kit for Illumina. Library size and quality were assessed using an HS-DNA chip on an Agilent Bioanalyzer 2100 (Agilent Technologies, Palo Alto, CA). Library concentrations were measured by quantitative PCR with KAPA Illumina Library Quantification Kits (KAPA Biosystems, MA, USA) on an ABI 7300 Plus machine (Thermo Fisher Scientific, MA, USA). Each library was sequenced with 150 bp paired-end reagents on Illumina platforms (Illumina, Inc., San Diego, CA). Amplicon metagenomics was adopted to explore the gut bacterial stability of the frequently donating donors over time. Samples were consecutively (with an interval of one week) collected from 16 donors from March 2018 to August 2019. They were then amplified using the 16S V4 primer set: 515F (5′-GTGCCAGCMGCCGCGGTAA-3′) and 806R (5′-GGACTACNVGGGTWTCTAAT-3′), following the methods reported [47], and sequenced on the Illumina MiniSeq with 150 bp paired-end reagents. All the protocols were followed according to the respective manufacturer’s instructions.

### Bioinformatic analyses, microbiota evaluation, and stability

For shotgun metagenomics, raw sequencing data and human genome reads were trimmed using KneadData tool (https://huttenhower.sph.harvard.edu/kneaddata), set at default parameters. Taxonomic profiles were generated by MetaPhlan2 [48] with default parameters, except for “stat_q: 0.0” to obtain taxa as many as possible. Additional metagenomic data of 78 donors and healthy subjects from four published studies [49–52], were used as references for downstream analyses. A total of 13 harmful bacterial taxa (Table 2), and microbial richness were taken into account to build a donor microbial evaluation index (DoMEI). Each beneficial taxon scored one point if its abundance was greater than the median of the abundance of the 78 donors; otherwise, it received a negative point (Table 2). Similarly, the harmful taxon earned one point when its abundance was less than the median; otherwise, it received a negative point. Likewise, microbial richness scored five points if it was greater than the median; otherwise, it scored zero points. The sum of each feature was tabulated as the DoMEI score of the donor. According to this scoring, the DoMEI values theoretically range from − 29 to 34. The scores of our candidate donors were between 5 and 26, and the cutoff value for appropriate donors was recommended to 10. Second, donors were further assessed in terms of 13 clinical pathogens (Table 2). The relative abundance of each pathogen was checked whether more than 0.01%.

**Table 2:** Bacterial taxa of donor microbial evaluation index (DoMEI)

Beneficial bacteria	Harmful bacteria	Clinical pathogens
*Bifidobacterium*	*Campylobacter*	*Helicobacter pylori*
*Lactobacillus acidophilus*	*Haemophilus*	*Clostridium difficile*
*Clostridium butyricum*	*Veillonella*	*Vibrio cholerae*
*Akkermansia muciniphila*	*Enterobacter*	*Listeria monocytogenes*
*Propionibacterium freudenreichii*	*Aeromonas*	*Campylobacter coli*
*Lactobacillus helveticus*	*Pseudomonas aeruginosa*	*Staphylococcus aureus*
*Faecalibacterium prausnitzii*	*Klebsiella*	*Rotavirus*
*Lactobacillus reuteri*	*Desulfovibrio*	*Norovirus*
*Pediococcus acidilactici*	*Methanobrevibacter mithii*	*Mastadenovirus*
*Enterococcus hirae*	*Fusobacterium nucleatum*	*Atadenovirus*
*Megamonas*	*Mycobacterium tuberculosis*	*Shigella*
*Odoribacter*	*Escherichia coli*	*Salmonella*
*Citrobacter*	*Listeria*	*Yersinia*
*Butyricimonas*		
*Alistipes*		
*Bacteroides thetaiotaomicron*		

For 16S amplicon metagenomics, raw paired-end reads were assembled using FLASH [53]. Operational taxonomic units (OTU) clustering at 97% similarity was performed using USEARCH [54]. Representative sequences were annotated against the Silva database [55] for taxonomic classification by the Ribosomal database project (RDP) Classifier [56]. Each sample was rarefied to 24598 reads. Ecological diversity estimates and distance were calculated using the vegan package [57] and visualised using the ggplot2 package [58] in R 3.5.3 [59]. Alpha diversity mainly reflects the diversity within the sample [60]. We use the richness and Shannon indices to reflect the richness of the sample from the qualitative and quantitative levels, respectively. Beta diversity is an indicator to measure the similarity of microbial composition between samples. Through the calculation of the Bray–curtis distance [61], a quantitative measure of community differences, the differences between groups are displayed based on NMDS analysis [62]. Significance was tested using the Kruskal Wallis test or PERMANOVA with 999 permutations using vegan package [57].

## Supplementary Information

**Additional file 1: Appendix S1.** Online prescreening survey for stool donor screening.

**Additional file 2: Appendix S2.** Clinical assessment for stool donor screening.

**Additional file 3: Figure S1.** The top 30 dominant genera of the 16 frequently-donating donors.

**Additional file 4:** **Table S1. **Reasons for candidate donor exclusion (Stage 1- Online prescreening survey).

**Additional file 5:** **Table S2. **Reasons for candidate donor exclusion (Stage 2 – Clinical assessment).

**Additional file 6:** **Table S3. **Reasons for candidate donor exclusion (Stage 3-Gastrointestinal pathogens screening).

**Additional file 7: Table S4**. Reasons for candidate donor exclusion (Stage 4 – Blood screening).

**Additional file 8:** **Table S5.** Summary of samples, sampling days, number of OTUs, and the count of samples in phylum, class, order, family, genus.

## Data Availability

The raw sequencing data have been deposited in SRA under the accession number PRJNA664544.
