# Supplementary material for: A comprehensive approach to stool donor screening for faecal microbiota transplantation in China
Source: Microb Cell Fact. 2021 Nov 27;20:216. doi: 10.1186/s12934-021-01705-0 (PMC8626716; doi:10.1186/s12934-021-01705-0)
Supplement: Supplementary file 1 — Additional file 1: Appendix S1. Online prescreening survey for stool donor screening. [file 12934_2021_1705_MOESM1_ESM.docx]

**Additional file 1**

**Online prescreening survey for stool donor screening**

1. Name
2. ID number
3. Phone number
4. Gender
5. Age (year)
6. Height (cm)
7. Weight (kg)
8. Address
9. Educational background
10. Have you been a stool donor for faecal microbiota transplantation (FMT) previously? ○Yes ○No
11. Do you smoke?

○Yes ○No

1. Do you drink alcohol?

○Yes ○No

1. How often do you exercise?

○ less than 3 times a week ○ 3-6 times a week ○ every day

1. How long do you sleep every night?

○less than 7 hours ○more than 9hours ○about 8 hours

1. How often do you eat cakes, chocolate or sweets?

○every day ○often ○seldom

1. How often do you eat fruit and vegetables?

○every day ○often ○seldom

1. Do you stay up late in normal times? (Later than 12 PM)

○Never ○Occasionally ○Sometimes ○Often

1. Is there a history of medication in nearly 6 months (e.g. antibiotic, PPI, corticosteroids)?

○Yes ○No

1. Is there a history of recurrent gastrointestinal symptom in the last 3 months? (e.g. constipation, diarrhea, bloating or abdominal pain, bloody stool, jaundice, etc.)

○Yes ○No

1. Is there a history of infectious disease risk in the last 6 months? (e.g. acupuncture, tattoos, piercings, drug-taking, high-risk sex)

○Yes ○No ○Unknown

1. Are certain inheritable diseases more prevalent in your family? (e.g. colorectal cancer, inflammatory bowel disease, etc.)

○Yes ○No ○Unknown

1. Is there a history of disease? (e.g. Malignancy history, musculoskeletal/pain syndrome, gynecological condition, neurological disease, gastrointestinal disease, autoimmune disease, cardiovascular/metabolic disease, diabetes, hypertension, atopy, asthma, allergies)

○Yes ○No

1. Did you travel to countries with a higher infectious disease risk in the last 6 months?

○Yes ○No

1. Is there a history of mental disease (e.g. depression, anxiety)?

○Yes ○No

1. Have you ever had sexual contact with a man? (for men)

Have you ever had sexual contact with a bisexual or homosexual man? (for women)

○Yes ○No

1. Do you have a new sexual partner with whom you have commenced sexual relations within the last 12 months?

○Yes ○No

1. While visiting another country (for work or vacation), have you ever had sexual contact with people originating from that country?

○Yes ○No

1. Have you ever had sexual contact with someone who turned out to be infected with HIV, HTLV, Hepatitis, or Syphilis?

○Yes ○No

1. Have you been to a high-risk area in a SARS-­CoV-2 outbreak in the last 14 days? (New addition)

○Yes ○No

1. Have you been injected the SARS-­CoV-2 vaccine? (New addition)

○Yes ○No
