# Supplementary material for: A comprehensive approach to stool donor screening for faecal microbiota transplantation in China
Source: Microb Cell Fact. 2021 Nov 27;20:216. doi: 10.1186/s12934-021-01705-0 (PMC8626716; doi:10.1186/s12934-021-01705-0)
Supplement: Supplementary file 2 — Additional file 2: Appendix S2. Clinical assessment for stool donor screening. [file 12934_2021_1705_MOESM2_ESM.docx]

**Additional file 2**

**Clinical assessment for stool donor screening**

1. Check the essential information and online prescreening survey.
2. Have you ever been rejected as a blood donor? If yes, why?
3. Have you ever been exposured with HIV, HAV, HBV, HCV infection (e.g. blood transfusion, accidental needle stick injuries, blood exposure or received blood products, etc.) in the last 12 months? If yes, when?
4. Have you ever had intravenous drug, incarceration, tattoo, piercing, acupuncture within previous 6 months?
5. Has Creuzfeldt Jakob’s disease ever occurred in your family?
6. Are certain inheritable diseases more prevalent in your family? If yes, which one?
7. Do you have any family members with intestinal cancer or polyps? If yes, in which relatives?
8. Have you ever had an operation? If yes, which, when and where?
9. Have you been to a tropical country in the last 6 months? (e.g. areas of high risk of travelers’ diarrhea). If yes, where and when?
10. Have you ever visited a medical specialist in the last 12 months? If yes, when, and for what reason.
11. Have you ever had an infectious disease? (e.g. Trypanosomiasis, Tuberculosis, Herpes, Malaria). If yes, which one? in what year?
12. Have you received vaccinations (not immunoglobulin’s)? For Hepatitis A? For Hepatitis B? If yes, was your antibody response for hepatitis B vaccination measured and adequate?
13. Have you ever had sexual practices associated with high risk of acquiring infectious diseases in last 12 months? (e.g. sexual contact with homosexual, someone infected with HIV, HTLV, Hepatitis, or Syphilis, work as a prostitute, etc.)
14. Is there a history of disease? If yes, when? what diseases? (e.g. Malignancy history, musculoskeletal/pain syndrome, gynecological condition, neurological disease, gastrointestinal disease, autoimmune disease, cardiovascular/metabolic disease, diabetes, hypertension, atopy, asthma, allergies, etc.)
15. Is there a history of communicable disease? (e.g. HIV, HAV, HBV, HCV, Syphilis, etc.)
16. Have you had the symptoms of infection in the previous 4 weeks? (e.g. fever, vomiting, diarrhea, etc.)
17. Have you had the vaccinations, injections or contact with a recipient in the previous 8 weeks? (e.g. smallpox vaccine, etc.)
18. Do you live with members who have active gastrointestinal infection? If yes, when? which ones?
19. Have you used medications in the previous 6 months? If yes, why and when? (e.g. PPI, traditional Chinese medicine, receiving growth hormone, an experimental medicine, immunomodulatory therapy, chemotherapy, etc.)
20. Have you used antibiotics in the past 3 months? Have you used antibiotics in the last year? If yes, when? What antibiotics?
21. Is there a history of intrinsic gastrointestinal disease (e.g. inﬂammatory bowel disease, irritable bowel syndrome, chronic constipation, gastrointestinal malignancy, prior major gastrointestinal surgery, procedure, etc.)?
22. Do you have the digestive system symptoms (e.g. difficulty defecating, abdominal cramps, flatulence, etc.)?
23. Do you have regular bowel movements? On average, how many bowel movements do you have in a day?
24. Do you have haemorrhoids? Have you ever had blood in your stools? If yes, were additional investigations performed? What were the results?
25. Do you have oral diseases? Dental check-up. (e.g. Caries, periodontal diseases, mucosal diseases or oral cancer).
26. Psychological assessment. (e.g. Hamilton Anxiety Rating Scale and Hamilton Depression Rating Scale).
27. Logistics issue. (e.g. unable to donate regularly, distance to donor facility).
28. Lifestyle questionnaire. (e.g. exercise, food frequency, etc.)
29. Social history. (e.g. smoking, drinking, etc.)
30. Do you have irregular menstruation or abnormal vaginal discharge? (for women)
31. Have you been to a high-risk area in a SARS-­CoV-2 outbreak in the last 14 days?
32. Have you been injected the SARS-­CoV-2 vaccine? If yes, when?
