# Supplementary material for: A comprehensive approach to stool donor screening for faecal microbiota transplantation in China
Source: Microb Cell Fact. 2021 Nov 27;20:216. doi: 10.1186/s12934-021-01705-0 (PMC8626716; doi:10.1186/s12934-021-01705-0)
Supplement: Supplementary file 3 — Additional file 3: Figure S1. The top 30 dominant genera of the 16 frequently-donating donors. [file 12934_2021_1705_MOESM3_ESM.docx]

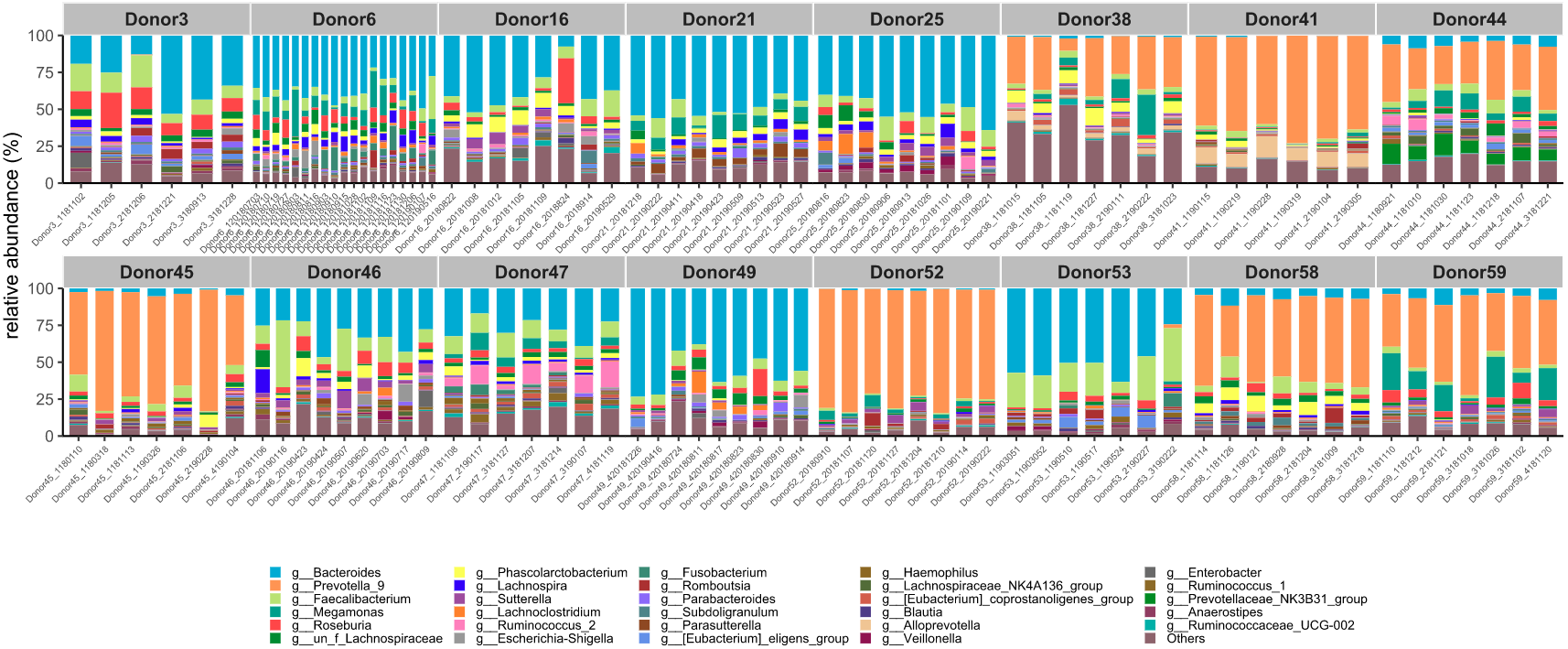


Additional file 3: Figure S1. The top 30 dominant genera of the 16 frequently-donating donors. Bacteria genera are represented in different colors. The horizontal axis represents the sample number and the vertical axis represents the relative abundance of the genera
