# Supplementary material for: A comprehensive approach to stool donor screening for faecal microbiota transplantation in China
Source: Microb Cell Fact. 2021 Nov 27;20:216. doi: 10.1186/s12934-021-01705-0 (PMC8626716; doi:10.1186/s12934-021-01705-0)
Supplement: Supplementary file 4 — Additional file 4: Table S1. Reasons for candidate donor exclusion (Stage 1- Online prescreening survey). [file 12934_2021_1705_MOESM4_ESM.docx]

| **Additional file 4: Table S1. Reasons for candidate donor exclusion (Stage 1- Online prescreening survey)** | | | | | | |  |
| --- | --- | --- | --- | --- | --- | --- | --- |
| **Reason for exclusion** | **Xiamen** | | **Guangzhou** | | **Sum total** | |  |
|  | **Frequency(n)** | **Excluding rate (%)** | **Frequency(n)** | **Excluding rate (%)** | **Frequency(n)** | **Excluding rate (%)** |  |
|  |  |  |  |  |  |  |  |
| Social history (smoking or drinking) | 197 | 14.04% | 89 | 13.32% | 286 | 13.81% |  |
| Logistics (e.g. unable to donate regularly, distance to donor facility) | 177 | 12.62% | 78 | 11.68% | 255 | 12.31% |  |
| Body mass index (>28 or <18.5kg/m2) | 109 | 7.77% | 58 | 8.68% | 167 | 8.06% |  |
| Medication history in nearly 6 months (e.g. antibiotic, PPI, corticosteroids ) | 85 | 6.06% | 54 | 8.08% | 139 | 6.71% |  |
| Infectious disease risk (e.g. acupuncture, tattoos, piercings, acupuncture) | 61 | 4.35% | 44 | 6.59% | 105 | 5.07% |  |
| Recurrent gastrointestinal symptoms (e.g. constipation, diarrhea, bloating or abdominal pain) | 68 | 4.85% | 26 | 3.89% | 94 | 4.54% |  |
| Family history (e.g. colorectal cancer，inflammatory bowel disease) | 55 | 3.92% | 26 | 3.89% | 81 | 3.91% |  |
| Disease history (e.g. autoimmune disease, diabetes, atopy, asthma, allergies) | 40 | 2.85% | 38 | 5.69% | 78 | 3.77% |  |
| Lifestyle questionnaire survey（Almost never exercise） | 50 | 3.56% | 19 | 2.84% | 69 | 3.33% |  |
| **Other** | | | | | | |  |
| Travel history (e.g. travel to countries with a higher infectious disease risk) | 41 | 2.92% | 8 | 1.20% | 49 | 2.37% |  |
| Mental health condition (e.g. depression, anxiety) | 15 | 1.07% | 17 | 2.54% | 32 | 1.55% |  |
| Age (>40 years or <18 years or Not provided) | 22 | 1.57% | 7 | 1.05% | 29 | 1.40% |  |
| High risk sexual behavior | 7 | 0.50% | 3 | 0.45% | 10 | 0.48% |  |
|  | 927 | 66.07% | 467 | 69.91% | 1394 | 67.31% |  |
| **Enter stage 2 (n)** | 476 |  | 201 |  | 677 |  |  |
