# Supplementary material for: A comprehensive approach to stool donor screening for faecal microbiota transplantation in China
Source: Microb Cell Fact. 2021 Nov 27;20:216. doi: 10.1186/s12934-021-01705-0 (PMC8626716; doi:10.1186/s12934-021-01705-0)
Supplement: Supplementary file 5 — Additional file 5: Table S2. Reasons for candidate donor exclusion (Stage 2 – Clinical assessment). [file 12934_2021_1705_MOESM5_ESM.docx]

| **Additional file 5: Table S2. Reasons for candidate donor exclusion (Stage 2 – Clinical assessment)** | | | | | | |  |
| --- | --- | --- | --- | --- | --- | --- | --- |
| **Reason for exclusion** | **Xiamen** | | **Guangzhou** | | **Sum total** | |  |
|  | **Frequency(n)** | **Excluding rate (%)** | **Frequency(n)** | **Excluding rate (%)** | **Frequency(n)** | **Excluding rate (%)** |  |
|  |  |  |  |  |  |  |  |
| Lost to follow-up (e.g. did not attend invited assessment) | 78 | 16.39% | 59 | 29.35% | 137 | 20.24% |  |
| Medications & supplements (e.g. having antibiotic, PPI，or receiving growth hormone, or receiving an experimental medicine) | 60 | 12.61% | 21 | 10.45% | 81 | 11.96% |  |
| Social history (smoking or drinking) | 37 | 7.77% | 14 | 6.97% | 51 | 7.53% |  |
| Disease history (e.g. Malignancy history，musculoskeletal/pain syndrome, gynecological condition, neurological disease, gastrointestinal disease, autoimmune disease, cardiovascular/metabolic disease, diabetes, hypertension, atopy, asthma, allergies,etc.) | 34 | 7.14% | 8 | 3.98% | 42 | 6.20% |  |
| Infectious disease history (e.g. hepatitis B) | 14 | 2.94% | 12 | 5.97% | 26 | 3.84% |  |
| Oral screening (Caries, periodontal diseases, mucosal diseases and oral cancer) | 15 | 3.15% | 7 | 3.48% | 22 | 3.25% |  |
| Hamilton Anxiety Rating Scale (>7 score) | 43 | 9.03% | 9 | 4.48% | 52 | 7.68% |  |
| Hamilton Depression Rating Scale (>7 score) | 19 | 3.99% | 6 | 2.99% | 25 | 3.69% |  |
| **Other** | | | | | | |  |
| Sexual history (high risk) | 12 | 2.52% | 5 | 2.49% | 17 | 2.51% |  |
| Logistics (e.g. unstable dwelling，unable to donate regularly, distance to donor facility) | 9 | 1.89% | 2 | 1.00% | 11 | 1.62% |  |
| Intellingence Quotient Test (accurate rate <40%) | 5 | 1.05% | 5 | 2.49% | 10 | 1.48% |  |
| Emotional Intelligence Test (<90 score) | 7 | 1.47% | 4 | 1.99% | 11 | 1.62% |  |
| Family history (e.g. colorectal cancer, inflammatory bowel disease) | 7 | 1.47% | 1 | 0.50% | 8 | 1.18% |  |
| Restrictive diet (e.g. gluten free diet) | 3 | 0.63% | 2 | 1.00% | 5 | 0.74% |  |
| Surgical history (e.g. digestive system surgery) | 3 | 0.63% | 1 | 0.50% | 4 | 0.59% |  |
| Travel history (e.g. travel to countries with a higher infectious disease risk) | 1 | 0.21% | 2 | 1.00% | 3 | 0.44% |  |
| Body mass index (>30 kg/m2 or <18kg/m2) | 2 | 0.42% | 1 | 0.50% | 3 | 0.44% |  |
| Health condition per physician discretion | 2 | 0.42% | 1 | 0.50% | 3 | 0.44% |  |
| Abnormal vital sign | 1 | 0.21% | 0 | 0.00% | 1 | 0.15% |  |
|  | 351 | 73.74% | 160 | 79.60% | 511 | 75.48% |  |
| **Enter stage 3 (n)** | 125 |  | 41 |  | 166 |  |  |
