# Supplementary material for: A comprehensive approach to stool donor screening for faecal microbiota transplantation in China
Source: Microb Cell Fact. 2021 Nov 27;20:216. doi: 10.1186/s12934-021-01705-0 (PMC8626716; doi:10.1186/s12934-021-01705-0)
Supplement: Supplementary file 6 — Additional file 6: Table S3. Reasons for candidate donor exclusion (Stage 3-Gastrointestinal pathogens screening). [file 12934_2021_1705_MOESM6_ESM.docx]

| **Additional file 6: Table S3. Reasons for candidate donor exclusion (Stage 3-Gastrointestinal pathogens screening)** | | | | | |
| --- | --- | --- | --- | --- | --- |
| **Reason for exclusion** | | **Xiamen** | **Guangzhou** | **Sum total** | **Total excluding rate (%)** |
|  |  | **Frequency(n)** | **Frequency(n)** | **Frequency(n)*** |  |
|  |  |  |  |  |  |
| Lost to follow-up (e.g. did not complete the screening tests) | | 12 | 2 | 14 | 8.43% |
| Fecal occult blood test | | 3 | 1 | 4 | 2.41% |
| *Helicobacter pylori* | | 36 | 8 | 44 | 26.51% |
| Intestinal parasites | Fecal egg, cyst, microsporidia and parasites | 2 | 2 | 4 | 1.81% |
|  | *Blastocystis hominis* | 1 | 0 | 1 | 0.00% |
|  | Strongyloides stercoralis | 0 | 0 | 0 | 0.00% |
|  | *Cyclospora/Isospora* | 0 | 0 | 0 | 0.00% |
|  | *Giardia* | 0 | 0 | 0 | 0.00% |
|  | *Cryptosporidium* | 0 | 0 | 0 | 0.00% |
| Intestinal pathogenic virus | Rotavirus | 3 | 1 | 4 | 2.41% |
|  | Norovirus | 0 | 0 | 0 | 0.00% |
|  | Adenovirus | 0 | 0 | 0 | 0.00% |
| Intestinal pathogenic bacteria | *C. difficile* | 0 | 0 | 0 | 0.00% |
|  | *Amoeba dysenteriae* | 0 | 0 | 0 | 0.00% |
|  | Shiga toxin with reflex to O157 | 0 | 0 | 0 | 0.00% |
|  | *Salmonella/Shigella* | 0 | 0 | 0 | 0.00% |
|  | *Vibrio cholera O1 and O139* | 0 | 0 | 0 | 0.00% |
|  | Listeria monocytogenes | 0 | 0 | 0 | 0.00% |
|  | Escherichia coli O157 H7 | 0 | 0 | 0 | 0.00% |
|  | Yersinia | 0 | 0 | 0 | 0.00% |
|  | *Campylobacter* | 0 | 0 | 0 | 0.00% |
| Intestinal drug-resistance bacteria | Methicillin-Resistant *Staphylococcus aureus* | 0 | 0 | 0 | 0.00% |
|  | Vancomycin-Resistant *Enterococci* | 0 | 0 | 0 | 0.00% |
|  | Carbapenem-Resistant *Enterobacteriaceae* | 0 | 0 | 0 | 0.00% |
|  |  | 57 | 14 | 71 | 41.57% |
| **Enter stage 4 (n)** | | 69 | 28 | 97 |  |

*There was a total of 71 abnormal stool screening test results among 166 candidate donors (i.e. some candidate donors had more than one abnormal test result)
