# Supplementary material for: A comprehensive approach to stool donor screening for faecal microbiota transplantation in China
Source: Microb Cell Fact. 2021 Nov 27;20:216. doi: 10.1186/s12934-021-01705-0 (PMC8626716; doi:10.1186/s12934-021-01705-0)
Supplement: Supplementary file 7 — Additional file 7: Table S4. Reasons for candidate donor exclusion (Stage 4 – Blood screening). [file 12934_2021_1705_MOESM7_ESM.docx]

| **Additional file 7: Table S4. Reasons for candidate donor exclusion (Stage 4 – Blood screening)** | | | | | |  |
| --- | --- | --- | --- | --- | --- | --- |
| **Reason for exclusion** | | **Xiamen** | **Guangzhou** | **Sum total** | **Total excluding rate(%)** |  |
|  |  | **Frequency(n)** | **Frequency(n)** | **Frequency(n)*** |  |  |
|  |  |  |  |  |  |  |
| Lost to follow-up (e.g. did not complete the screening tests) | | 5 | 1 | 6 | 6.19% |  |
| Infectious pathogen | Syphilis serum antibody test (+) | 1 | 0 | 1 | 6.19% |  |
|  | HTLV I/II | 0 | 0 | 0 | 0.00% |  |
|  | HAV | 0 | 0 | 0 | 0.00% |  |
|  | HBV | 4 | 2 | 6 | 0.00% |  |
|  | HCV | 0 | 0 | 0 | 0.00% |  |
|  | HEV | 0 | 0 | 0 | 0.00% |  |
|  | HIV | 0 | 0 | 0 | 0.00% |  |
| Abnormal complete blood count | Low white blood cell count | 1 | 1 | 2 | 6.19% |  |
|  | Low hemoglobin | 2 | 2 | 4 | 0.00% |  |
|  | Elevated eosinophils | 1 | 3 | 4 | 0.00% |  |
|  | Other abnormal hematology or outside reference range | 1 | 0 | 1 | 0.00% |  |
| Abnormal liver function tests | Elevated bilirubin | 2 | 1 | 3 | 8.25% |  |
|  | Elevated AST | 2 | 1 | 3 | 0.00% |  |
|  | Elevated ALT | 2 | 1 | 3 | 0.00% |  |
|  | Low ALP | 1 | 1 | 2 | 0.00% |  |
|  | High gamma-glutamyl transpeptidase | 1 | 1 | 2 | 0.00% |  |
| Abnormal kidney function tests | Elevated blood creatinine | 1 | 1 | 2 | 2.06% |  |
|  | Elevated blood urea nitrogen | 1 | 1 | 2 | 0.00% |  |
|  | Elevated blood trioxypurine | 3 | 2 | 5 | 0.00% |  |
| Dyslipidemia | High cholesterolemia (CHOL) | 3 | 2 | 5 | 3.09% |  |
|  | High triglycerides (TG) | 2 | 2 | 4 | 0.00% |  |
|  | Elevated low density lipoprotein（LDL） | 0 | 0 | 0 | 0.00% |  |
| Blood glucose | | 0 | 1 | 1 | 0.00% |  |
| Elevated C-Reactive Protein levels | | 1 | 0 | 1 | 0.00% |  |
| Elevated dynamic ESR | | 0 | 0 | 0 | 0.00% |  |
|  |  | 34 | 23 | 57 | 31.96% |  |
| **Eligible donor** | | 45 | 21 | 66 |  |  |
| *There was a total of 57 abnormal serological screening test results among 31 candidate donors (i.e. some candidate donors had more than one abnormal test result) | | | | | |  |
|  |  |  |  |  |  |  |
|  |  |  |  |  |  |  |
