# Supplementary material for: A comprehensive approach to stool donor screening for faecal microbiota transplantation in China
Source: Microb Cell Fact. 2021 Nov 27;20:216. doi: 10.1186/s12934-021-01705-0 (PMC8626716; doi:10.1186/s12934-021-01705-0)
Supplement: Supplementary file 8 — Additional file 8: Table S5. Summary of samples, sampling days, number of OTUs, and the count of samples in phylum, class, order, family, genus. [file 12934_2021_1705_MOESM8_ESM.docx]

Additional file 8: Table S5. Summary of samples, sampling days, number of OTUs, and the count of samples in phylum, class, order, family, genus.

| Donor | Days | OTUs | Phylum | Class | Order | Family | Genus |
| --- | --- | --- | --- | --- | --- | --- | --- |
| Donor_49 | 266 | 250 | 10 | 16 | 24 | 43 | 130 |
| Donor_38 | 130 | 450 | 12 | 20 | 29 | 56 | 172 |
| Donor_16 | 280 | 391 | 11 | 19 | 26 | 51 | 161 |
| Donor_25 | 189 | 235 | 11 | 17 | 27 | 52 | 126 |
| Donor_21 | 160 | 220 | 11 | 18 | 26 | 43 | 123 |
| Donor_46 | 276 | 319 | 9 | 16 | 25 | 52 | 157 |
| Donor_3 | 106 | 294 | 11 | 17 | 23 | 49 | 168 |
| Donor_47 | 70 | 405 | 13 | 19 | 30 | 57 | 171 |
| Donor_45 | 440 | 278 | 12 | 18 | 25 | 48 | 134 |
| Donor_53 | 91 | 216 | 11 | 17 | 25 | 44 | 111 |
| Donor_58 | 115 | 243 | 9 | 14 | 22 | 40 | 121 |
| Donor_59 | 55 | 263 | 10 | 17 | 24 | 48 | 131 |
| Donor_41 | 74 | 219 | 8 | 14 | 18 | 36 | 133 |
| Donor_52 | 165 | 299 | 13 | 19 | 27 | 50 | 148 |
| Donor_44 | 91 | 346 | 10 | 16 | 24 | 48 | 138 |
| Donor_6 | 317 | 281 | 11 | 17 | 28 | 58 | 146 |
